# Probing the expression and adhesion of glycans involved in *Helicobacter pylori* infection

**DOI:** 10.1038/s41598-024-59234-w

**Published:** 2024-04-13

**Authors:** Daniel Sijmons, Simon Collett, Caroline Soliman, Andrew J. Guy, Andrew M. Scott, Lindy G. Durrant, Aaron Elbourne, Anna K. Walduck, Paul A. Ramsland

**Affiliations:** 1 School of Science, RMIT University, Melbourne, VIC 3000 Australia; 2 Department of Paediatrics, The University of Melbourne, Parkville, VIC 3010 Australia; 3 Department of Microbiology and Immunology, Peter Doherty Institute for Infection and Immunity, The University of Melbourne, Melbourne, VIC 3000 Australia; 4 ZiP Diagnostics, Collingwood, VIC 3066 Australia; 5 Olivia Newton-John Cancer Research Institute and School of Cancer Medicine, La Trobe University, Melbourne, VIC Australia; 6 Department of Molecular Imaging and Therapy, Austin Health and Faculty of Medicine, The University of Melbourne, Melbourne, VIC Australia; 7 Scancell Limited, University of Nottingham Biodiscovery Institute, Nottingham, UK; 8 Division of Cancer and Stem Cells, School of Medicine, University of Nottingham Biodiscovery Institute, Nottingham, UK; 9 Rural Health Research Institute, Charles Sturt University, Orange, NSW 2800 Australia; 10 Department of Immunology, Monash University, Melbourne, VIC 3004 Australia; 11 Department of Surgery, Austin Health, The University of Melbourne, Heidelberg, VIC 3084 Australia

**Keywords:** Microbiology, Bacteriology

## Abstract

*Helicobacter pylori* infects approximately half the human population and has an unusual infective niche of the human stomach. *Helicobacter pylori* is a major cause of gastritis and has been classified as a group 1 carcinogen by the WHO. Treatment involves triple or quadruple antibiotic therapy, but antibiotic resistance is becoming increasingly prevalent. *Helicobacter pylori* expresses certain blood group related antigens (Lewis system) as a part of its lipopolysaccharide (LPS), which is thought to assist in immune evasion. Additionally, *H. pylori* LPS participates in adhesion to host cells alongside several adhesion proteins. This study profiled the carbohydrates of *H. pylori* reference strains (SS1 and 26695) using monoclonal antibodies (mAbs) and lectins, identifying interactions between two carbohydrate-targeting mAbs and multiple lectins. Atomic force microscopy (AFM) scans were used to probe lectin and antibody interactions with the bacterial surfaces. The selected mAb and lectins displayed an increased adhesive force over the surface of the curved *H. pylori* rods. Furthermore, this study demonstrates the ability of anti-carbohydrate antibodies to reduce the adhesion of *H. pylori* 26695 to human gastric adenocarcinoma cells via AFM. Targeting bacterial carbohydrates to disrupt crucial adhesion and immune evasion mechanisms represents a promising strategy for combating *H. pylori* infection.

## Introduction

*Helicobacter pylori* (*H. pylori*) is a gram-negative microorganism that infects approximately 50% of the human population and can cause a variety of pathologies including chronic gastritis and gastric polyps in up to 15% of cases, and gastric malignancies, such as gastric adenocarcinoma, in 1–2% of cases^1–4^. An important step in *H. pylori* infection involves adhesion of the microorganism to the gastric epithelium and the gastric mucosa. Boren et al. first reported that this process was associated with the histo-blood group antigen Lewis B (Le^b^) as well as sialyl-Lewis X (SLe^x^)^5–7^. Adhesion is mediated by outer membrane proteins (OMPs) such as the blood group binding adhesin (BabA) and sialic acid binding adhesin (SabA) which bind to Le^b^ and SLe^x^ respectively^6–11^. In addition, *H. pylori* has also been found to express Lewis antigens as a part of its lipopolysaccharide (LPS), thought to be a form of molecular mimicry allowing the bacterium to hide from the immune system by blending in with the gastric epithelia^12,13^. LPS is a biomolecule consisting of lipid and polysaccharide located in the outer membrane of Gram-negative bacteria. *Helicobacter pylori* LPS plays a synergistic role with OMPs in binding to host gastric cells. Inhibition of LPS carbohydrate determinants has been shown to affect adherence of clinical isolates of *H. pylori* to human cells in culture^14,15^.

*Helicobacter pylori* LPS structure includes lipid A, core-oligosaccharide, and O antigen^16^. The O antigen contains a glucan group, a DD-heptan group and a highly conserved “trio” trisaccharide^16,17^. Variability of LPS occurs between strains, and due to bacterial phase variation, whereby *H. pylori* uses an on/off environmental sensor system to regulate biosynthetic genes that alter carbohydrate expression of the O antigen^18^. Several studies have examined the molecular structure of *H. pylori* glycans and LPS^12,16,19,20^. One such study used agglutination techniques to identify lectin reactivity, while another utilised a lectin microarray^14,19^. Silva et al.^19^ identified Le^x^ and Le^y^ as well as *N-*acetyllactosamine determinates on all *H. pylori* clinical isolates tested. Additionally, 1,3-d-galactans and blood group H-type 2 antigens were identified in some clinical isolates^19^. Chionh et al.^14^ examined if carbohydrates can inhibit binding *H. pylori* to human cell lines. Mannose, *N*-acetylgalactosamine, *N*-acetylglucosamine, fucose and sialic acid were significantly more effective at inhibiting cellular binding of *H. pylori* clinical isolates compared to mouse adapted strains^14^. The structural diversity and variation of the *H. pylori* LPS presents a challenge in establishing an accurate structure–function relationship between the *H. pylori* LPS and an infected host. Further structural studies can provide a better picture of the underlying molecular interactions of the *H. pylori* LPS as a part of its role in pathogenesis^19^. Additionally, the *H. pylori* LPS appears to play an important role in the adhesion of the bacteria to gastric cells, with disruption of the LPS significantly reducing the organism’s adhesion to gastric cells^21^.

The ability of atomic force microscopy (AFM) to measure mechanical and adhesive properties facilitates the characterisation of surface properties of microbial cells^22–26^. The peptidoglycan layer of live microbes such as *Escherichia coli* and *Pseudomonas aeruginosa* have been previously investigated using AFM in aqueous conditions^27^. AFM has also been used to measure the biophysical properties of bacteria such as *H. pylori* allowing for a deeper understanding of surface adhesion that can be applied to other protein-carbohydrate interactions for assessment in drug discovery^28^. For *H. pylori* specifically*,* AFM has been used to look at interactions between Le^b^ and *H. pylori* blood group binding adhesin (BabA). Based on the complexity of the interaction, Parreira et al. proposed a two-step model for ligand binding for the BabA-Le^b^ complex^29^.

This study aimed to profile glycans expressed by *H. pylori* using lectins and monoclonal antibodies (mAbs). The *H. pylori* strains SS1 and 26695 were selected as their glycans were expected to be adapted for efficient colonisation of mouse and human hosts, respectively. Proteins found to bind *H. pylori* were used in AFM experiments to directly measure the interactions of these proteins with *H. pylori* 26695. AFM was demonstrated to be useful in probing direct interactions between *H. pylori* 26695 and human gastric adenocarcinoma cells (AGS). Furthermore, antibody-binding to Le^y^ determinants in *H. pylori* 26695 LPS can reduce bacterial adhesion to AGS cells.

## Materials

### Immunofluorescence and ELISA

Lectins used for primary staining (Table 1) were from the biotinylated Lectin Kit 1 (BK-1000) and biotinylated Lectin Kit 2 (BK-2100) (Vector laboratories) and were used at a concentration of 10 μg/mL.

**Table 1:** Lectin binding specificities.

Lectin	Lectin abbreviation	Primary specificity
Concanavalin A	Con A	Mannose
Peanut agglutinin	PA	Galactose
*Ricinus communis* agglutinin	RCA	Galactose, *N*-acetylgalactosamine
Wheat Germ agglutinin	WGA	*N*-acetylglucosamine
Soybean agglutinin	SBA	*N*-acetylgalactosamine
*Ulex europaeus* agglutinin	UEA	Fucose
*Dolichos biflorus* agglutinin	DBA	*N*-acetylgalactosamine
*Sophora japonica* agglutinin	SJA	*N*-acetylgalactosamine
*Psathyrella velutina* lectin	PVL	*N*-acetylneuraminic
*Pisum sativum* agglutinin	PSA	Mannose
*Griffonia simplicifolia* lectin	GSL	*N*-acetylglucosamine
*Lens colinaris* agglutinin	LCA	Mannose
Succinylated wheat germ agglutinin	sWGA	*N*-acetylglucosamine
*Phaseolus vulgaris* erythroagglutinin	PHA-E	Complex type *N*-glycans containing bisecting *N-*acetylglucosamine or a 1,6-linked branch
Elder Bark Lectin	EBL	Sialic acid
*Euonymus europaeus* lectin	EEL	Human blood groups B and H
*Griffonia simplicifolia* lectin	GSL (isolectin B4)	Galactose
*Maackia amurensis* lectin	MAL	Galactose, Lactose

*All lectins provided by Vector laboratories.

The lectins used for immunofluorescence were Fluorescein Lectin Kit I (FLK-2100) and Fluorescein Lectin Kit II (FLK-3100) supplied by Vector laboratories. The panel of mAbs tested included FG27 and ch88.2 provided by Professor Lindy Durrant (Scancell UK, University of Nottingham UK), 15.101 provided by Emeritus Professor Mauro Sandrin (University of Melbourne, Australia), mu58 and mu3S193 provided by Professor Andrew Scott (ONJCRI), and 7LE, 2-25LE and LWY/1463 (Table 2) purchased from Abcam (Cambridge, UK). A nuclear stain (4′,6-diamidino-2-phenylindole, DAPI, Sigma/Merck) was used to ensure the presence of bacteria on the slide. The same mAbs used were for immunofluorescence and ELISA. All ELISAs were performed on 96-well NUNC plates (ThermoFisher, USA). The secondary antibodies used included goat anti-mouse IgG HRP ab6789 (abcam, USA) and goat anti-human IgG Fc secondary antibody HRP (ThermoFisher, USA) for ELISA, and goat anti-mouse IgG Alexa Fluor^tm^ 488 (ThermoFisher, USA) and goat anti-human IgG cross-absorbed secondary antibody Alexa Fluor^tm^ 488 (ThermoFisher, USA) for immunofluorescence. NeutrAvidin protein HRP (ThermoFisher, USA) was used for the detection of lectin binding.

**Table 2:** Monoclonal antibody binding specificities.

Monoclonal antibody	Specificity	Reference/provider
15.101	αGalactose	^30^
2-25LE	Lewis B	Abcam
CH88	Lewis A/X	^31^, Scancell
FG27	Lewis Y	Scancell
LWY/1463	Lewis Y	Abcam
mu58	Lewis B	Olivia Newton John Cancer Research Institute
mu3S193	Lewis Y	Olivia Newton John Cancer Research Institute^32^

### Atomic force microscopy

AFM was performed using a JPK nanowizard 4 (JPK BioAFM Business, Am Studio 2D, 12489 Berlin, Germany) using AFM RESPA-10 probes (Bruker, USA, nominal spring constant k_c_ = 0.1 N/m, nominal tip radius 8 nm, rectangular geometry, nominal cantilever length and width 45 µm and 5 µm, respectively, nominal resonance frequency 10 Hz) and tipless cantilevers AIO-TL-50 (Budget Sensors, USA—nominal spring constant = 0.2 N/m, nominal cantilever length and width 500 µm and 30 µm, nominal resonance frequency 15kHz) with fluorodish® (WPI, USA) glass bottomed petri dishes, thinly coated with poly-d-Lysine. AFM force measurements were obtained using a JPK nanowizard 4 (JPK BioAFM Business, Am Studio 2D, 12489 Berlin, Germany) operated in a liquid environment. Adhesion values were measured from the retraction profile of each force curve taking the minimum value of each profile, typically a negative value, which is then converted into a positive value to elucidate adhesive force. AFM data were processed using a combination of JPK software (https://www.jpk.com/), which allowed for the extraction of the single point adhesion measurements, and the Gwyddion 2.50 software package, which allowed for the processing of the AFM images and the extraction of the data for the percent frequency and cross section graphs.

## Methodology

### *Helicobacter pylori* culture and preparation of lysate

*Helicobacter pylori* strains SS1 (ATCC 43504) and 26695 (ATCC 700392) were revived from glycerol stocks and cultivated under microaerophilic conditions at 37 °C for 48–72 h on lysed horse blood agar (HBA). Bacteria were harvested and cultured overnight in 10 mL of brain heart infusion (BHI) broth supplemented with 10% heat-inactivated FCS (ThermoFisher, USA) (shaker incubator at 150 rpm, 37 °C). Bacteria were harvested by centrifugation and washed with 2 mL of phosphate buffered saline (PBS) and centrifuged at 5000 rpm for 10 min. The cells were then resuspended in 2 mL PBS and disrupted using microbeads and sonication. The presence and integrity of *H. pylori* proteins in the lysate of both strains was confirmed via gel electrophoresis and the lysate was then stored at − 80 °C.

### ELISA

The *H. pylori* lysate at a protein concentration of 10 µg/mL in carbonate–bicarbonate buffer pH 9.2 was used to coat the wells of 96-well NUNC plates. The ELISA was performed using a direct ELISA protocol; wells were blocked using 0.5% (m/v) poly(vinyl alcohol) for lectins and 1% goat serum for mAbs, and then incubated for 1 h at 37 °C and washed four times with tris-buffered saline (TBS) 0.05% Tween 20 (TBST) before testing with the panel of lectins and mAbs. After lectins and mAbs were added, the plate was incubated for an hour at 37 °C and washed four times with TBST. The secondary antibody for mAbs (goat anti-mouse IgG (abcam)) was added and the plate was incubated for 30 min at 37 °C, then washed again. Finally the substrate, 2,2-Azinobis [3-ethylbenzothiazoline-6-sulfonic acid]-diammonium salt (ABTS), was added developed at room temperature for 20 min. A pool of serum from mice that had been vaccinated against *H. pylori*^33^ was used as positive control, and a pool of serum from naive mice as a negative control. Each lectin and mAb was tested in four replicates over three separate ELISA tests.

### Immunofluorescence

Bacteria cultured from HBA plates were heat fixed onto Superfrost Plus glass slides (Thermo Fisher) for immunofluorescence (IF). IF was performed using a standard direct IF procedure. Slides were blocked with 1% goat serum for 20 min, then washed 3 times with PBS before addition of mAbs. The slides were incubated for 30 min and washed with PBS. The secondary (Alexa Fluor® 488) antibody was added and incubated for a further 30 min and the wash was repeated. Slides were counterstained with DAPI to visualise bacterial cells on the slides via detection of bacterial nucleic acid. A similar procedure was also used for lectins, with the addition of FITC-labelled lectins instead of mAbs, the slides were then incubated for 30 min and washed three times with PBS and counterstained with DAPI.

### AFM of bacterial cells

*Helicobacter pylori* 26695 was retrieved from solid culture and suspended in 1 mL PBS. Bacterial suspensions were incubated for 1 h at room temperature (RT) in a fluorodish® (WPI, USA) glass bottomed petri dish thinly coated with poly-d-Lysine, to allow attachment of *H. pylori* to the glass. Plates were then washed 3 times with PBS and re-wetted with 2 mL of PBS for probing in liquid. AFM RESPA-10 probes were treated by overnight incubation in either a 2 mg/mL solution of WGA or a 2 μg/mL solution of anti-Le^y^ (mu3S193) in PBS at 4 °C. The treated cantilever was then washed in PBS for 1 h at room temperature before use. JPK nanowizard 4 was operated in QI mode for all bacterial imaging experiments. QI mode allows for the successive collection of force *vs* distance curves, obtaining an individual force curve for each pixel. This provides height, adhesion and elasticity data for each individual location probed. RESPA-10 probes were used for all experiments and each cantilever was tuned using the thermal spectrum method. Images were collected at rates of 1.8–25.7 Hz with the rate of each collection adjusted to reduce background noise. The approach and retraction speeds were similarly adjusted and ranged between 175 and 240 μm/s over Z distances of 5–20 μm, with final images typically collected at 6–8 μm, at 128 × 128 pixels per frame. Set point values to ensure engagement of the cantilever typically fell between 1 and 3 nN of force.

### AFM of AGS cells

AGS cells (CRL-1739) were grown in RPMI 1640 medium (ThermoFisher, USA) with 10% foetal calf serum (FCS, ThermoFisher, USA) and 1% penicillin–streptomycin (ThermoFisher, USA) at 37 °C with 5% CO_2_ to 70% confluence and incubated on a fluorodish® overnight prior to measurement and used for AFM imaging within 4 h. *Helicobacter pylori* 26695 coated AIO-TL-50 tipless cantilevers were first incubated with polydopamine for 1 h and then pressed 3 times for 20-min intervals against a *H. pylori* 26695 prepared fluorodish® as described above, allowing for the *H. pylori* 26695 cells to coat the cantilever. AGS cells were first imaged with QI mode in the same manner as the above bacterial experiments and then adhesion measurements were taken using single point spectrometry with a retraction of 5 µm and were pressed against the cells for 10 s intervals.

### Statistical analysis and figure generation

All graphs and statistical data were stored and processed using GraphPad prism 9.5.0/10.0.0 and Microsoft Excel. IF images were captured using the DM 2500 Leica Fluorescence Microscope and processed using a combination of the Leica Aperio Laboratory Information Systems (LIS), Adobe Photoshop 2022 and ImageJ. Adobe Photoshop 2022 was also used to combine AFM images processed in Gwyddion 2.50 into figures.

## Results

ELISA and immunofluorescence were performed to characterise the glycan profile of *H. pylori* SS1 and 26695. Previous studies have identified the presence of Lewis antigens on *H. pylori*, although the exact Lewis type can vary between isolates. Additionally, of the lectins to be screened, only WGA and UEA had previously shown reactivity via agglutination assays^14,20^. For this study, we characterised a broader and different range of carbohydrates to previous studies and characterised the specific Lewis antigens present on the isolates of *H. pylori* SS1 and 26695.

### *Helicobacter pylori* SS1 displays a broader range of carbohydrates than 26695

**Figure 1:**
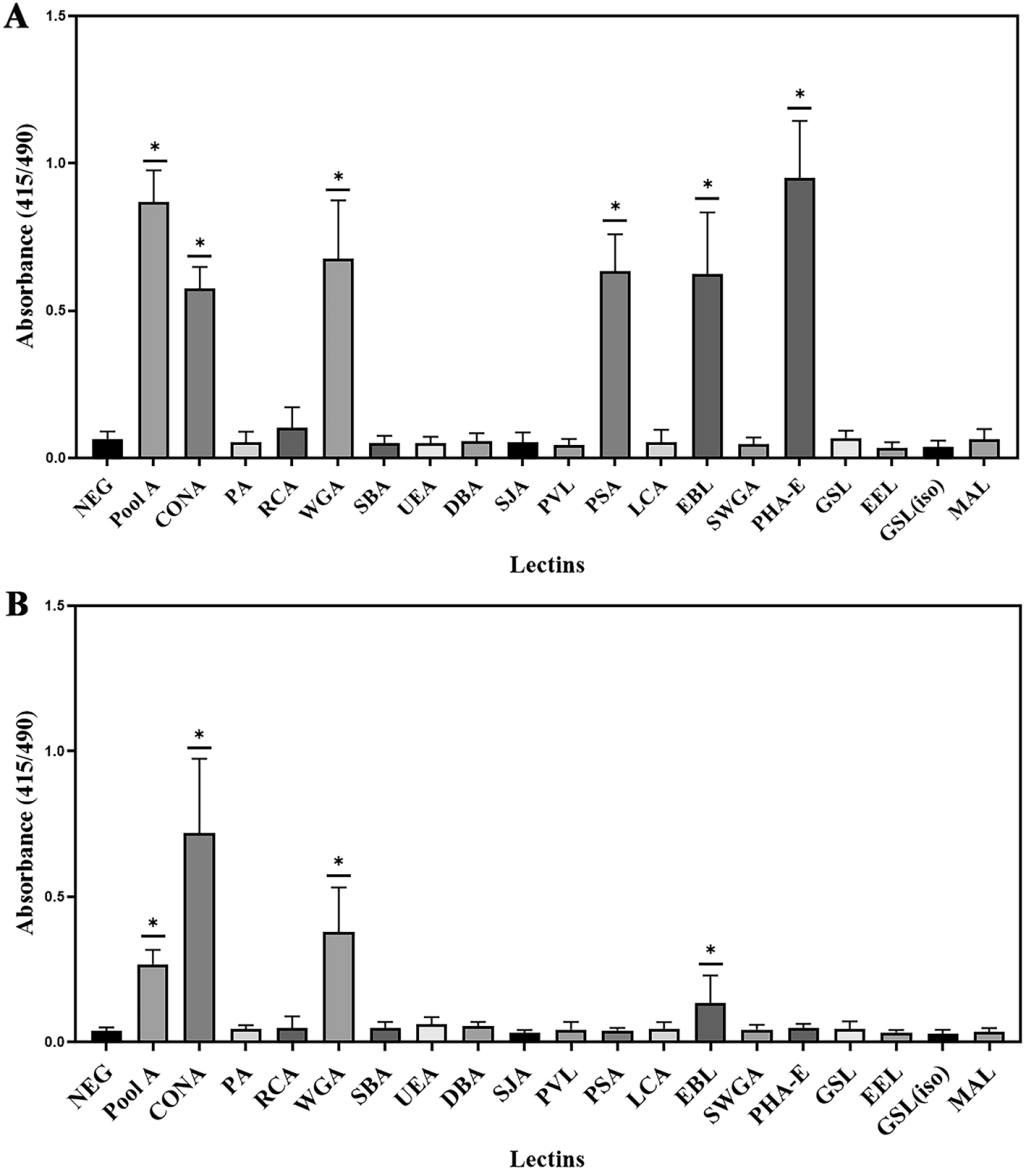
Lectin binding to *H. pylori* laboratory strains. (**A**) ELISA screening of the Lectin Array against *H. pylori* SS1 lysate (**B**) ELISA screening of the Lectin Array against *H. pylori* 26695 lysate. Pool A is a technical control (pooled *H. pylori* SS1 specific mouse serum). *p ≤ 0.05 by one-way ANOVA vs negative control. Each sample was run in quadruplicate in three separate assays, error bars represent standard deviation.

The glycans present in lysates of *H. pylori* (SS1 and 26695) were examined by ELISA against 18 lectins (Fig. 1). The SS1 lysate interacted strongly with 6 lectins including wheat germ agglutinin (WGA), concanavalin A (Con A), Elder bark lectin (EBL), *Phaseolus vulgaris* erythroagglutinin (PHA-E), and *Pisum sativum* agglutinin (PSA). This reactivity of lectins suggests the presence of glycans containing *N-*acetylglucosamine, mannose, sialic acid, and complex *N-*type glycans in the bacterial lysate of *H. pylori* SS1. Strain 26695 showed similarities to SS1, with both strains of *H. pylori* possessing *N*-acetylglucosamine and mannose based on the WGA and Con A binding. Interestingly, a wider array of lectin binding and higher absorbance values were observed for the SS1 strain compared to the 26695 strain. This finding suggests a wider glycan expression profile for SS1 in comparison to 26695. The intensity of the binding of serum pool A for *H. pylori* 26695 lysate was reduced in ELISA in comparison to the SS1 lysate. This probably reflects that the mice had been challenged with the mouse-adapted SS1 strain^34^ and therefore are likely to have had a more specific antibody response to the challenge strain.

### WGA and Con A interact with *H. pylori* SS1 and 26695 via Immunofluorescence and ELISA

**Figure 2:**
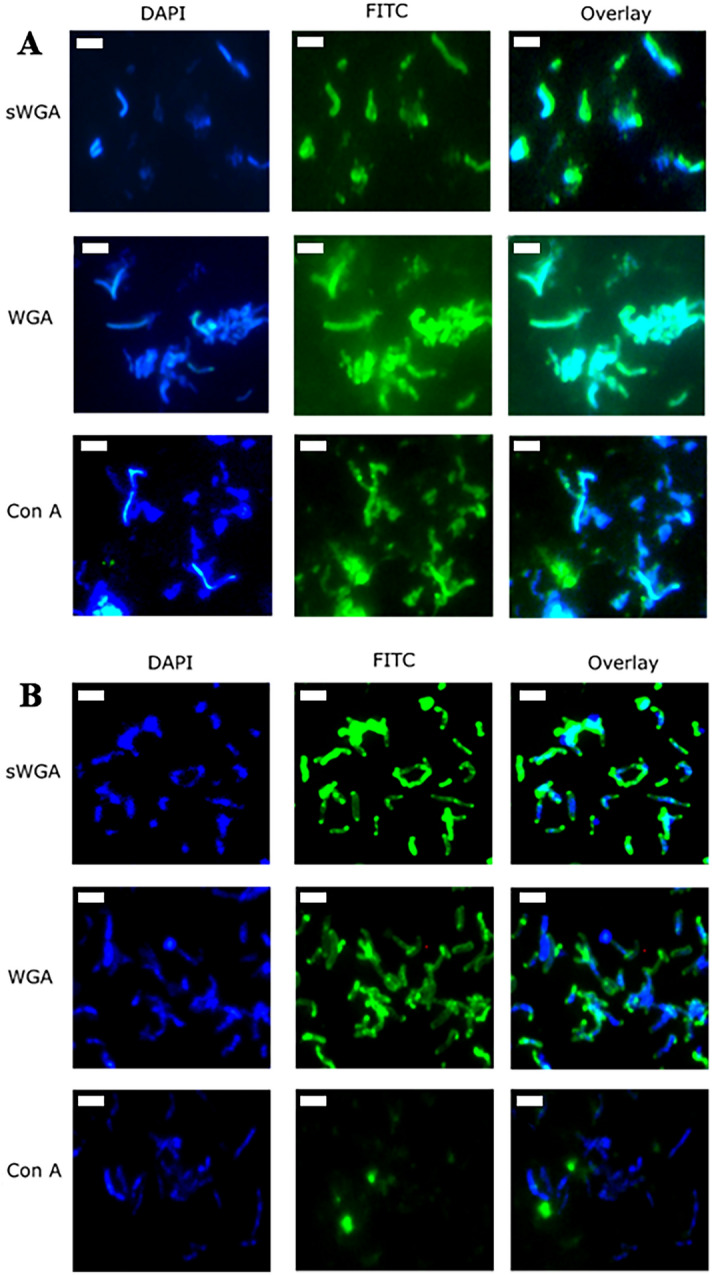
Lectin binding to surface of *H. pylori* laboratory strains. (**A**) Immunofluorescence images of lectins against *H. pylori* SS1. Con A WGA and sWGA appear to bind strongly (**B**) Immunofluorescence images of lectins against *H. pylori* 26695. Con A appears to bind moderately, while WGA and sWGA show a stronger binding in IF. The lectins PA, RCA, SBA, UEA, DBA, SJA, PVL, PSA, GSC, LCA. PHA-E, and GSL were also tested in immunofluorescence and showed no reactivity (Fig. S1). The white scale bar represents 2 μm.

Immunofluorescence staining using 18 FITC-labelled lectins against the SS1 strain revealed binding of sWGA, WGA and Con A on whole bacterial cells indicating the presence of *N-*acetylglucosamine and mannose on the bacterial surface (Fig. 2A). These results are similar to those obtained by ELISA; however, there were some key differences: the lectin PHA-E showed no reactivity against SS1 by IF (Fig. S1), and sWGA, which binds *N-*acetylglucosamine, showed reactivity via IF but did not show reactivity by ELISA. Note that sWGA did not bind to the lysate in ELISA, which is unusual since it binds to a similar glycan to WGA (which showed strong binding by ELISA). However, WGA is suggested to bind weakly to sialic acids, as well as *N-*acetylglucosamine, while sWGA does not, which is a possible explanation for the difference in binding seen here. PSA, PHA-E, and EBL did not show binding via IF but were positive in ELISA, indicating that these lectins may bind to internal polysaccharides which are exposed when the cells are lysed. Figure 2B shows the lectin IF assay of 26695, which is relatively consistent with the results obtained with SS1. The lectins Con A, WGA, and sWGA were found to interact with *H. pylori* 26695, although the binding of Con A was less specific than what was seen against SS1. The increased binding seen to SS1 in comparison with 26695 via the ELISA was less apparent in the IF. This may be a result of the limitation of the method in terms of quantification, as possible differences between the lysates used to prepare the ELISAs make direct comparison difficult, or it may be that SS1 has more internally expressed glycans which are not revealed by immunofluorescence on intact bacteria.

### Reactivity of mAbs and against *H. pylori* SS1 and 26695 via ELISA

**Figure 3:**
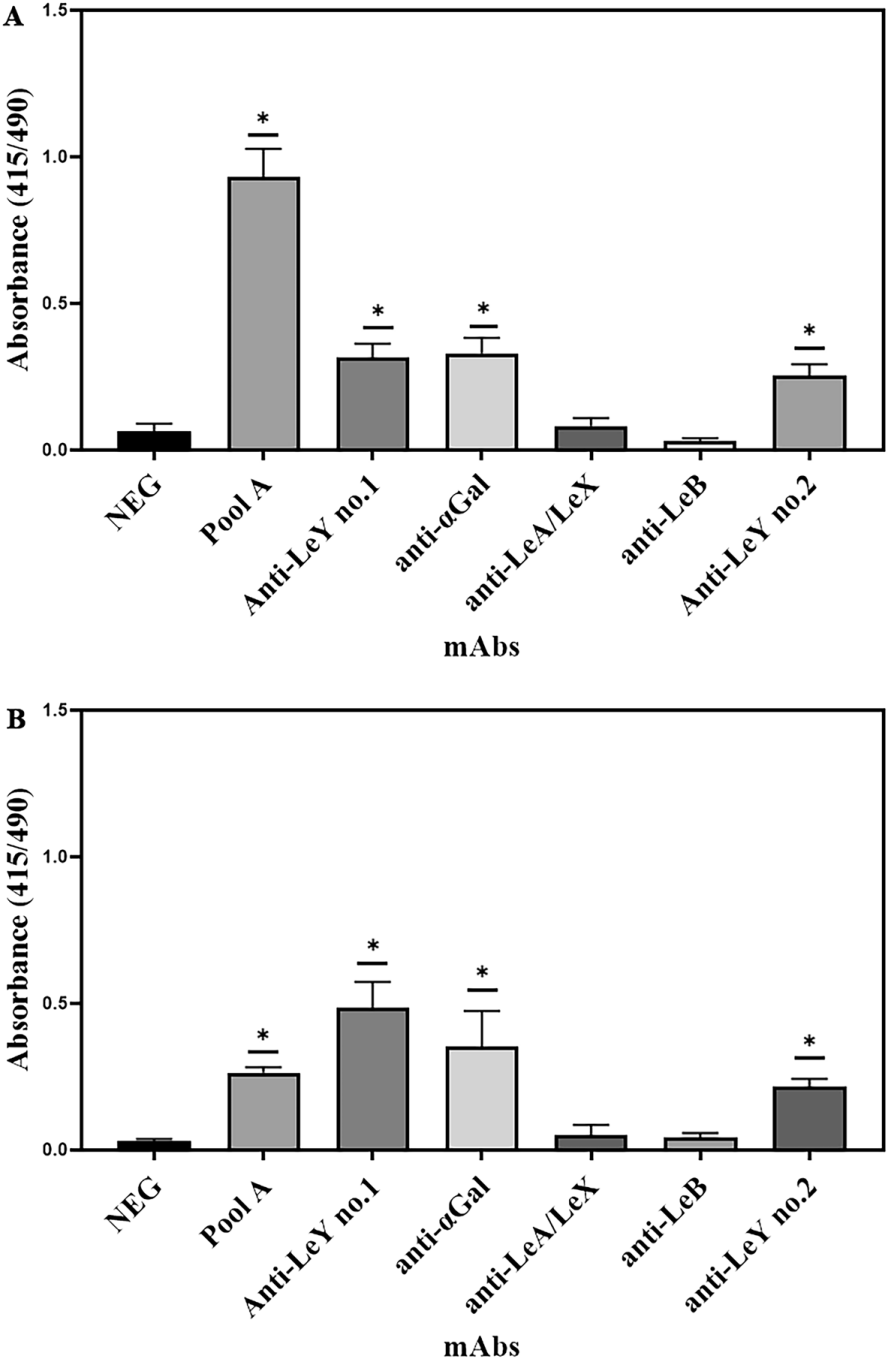
Carbohydrate binding mAbs interaction with *H. pylori* SS1 and 26695. (**A**) ELISA screen of Lewis antigen specific mAbs against *H. pylori* SS1 lysate. (**B**) ELISA of Lewis antigen antibodies against *H. pylori* 26695 lysate*.* Anti-αGal and both anti-Le^y^ mAbs showed reactivity to both SS1 and 26695 while the other mAbs showed no binding. *p ≤ 0.05 by one-way ANOVA vs neg.

Compared to lectins, mAbs generally recognise larger carbohydrate determinants and are generally more specific. Thus, we next examined the binding of *H. pylori* glycans with a panel of carbohydrate-binding mAbs. Of the mAbs tested, FG27, a mouse anti-Le^y^ IgG and 15.101, a mouse anti-αGal IgG showed reactivity against *H. pylori* SS1 via ELISA (Fig. 3). Three different anti-Le^y^ mAbs were shown to bind to the two *H. pylori* strains tested. The other anti-Lewis mAbs used did not show any indication of binding against *H. pylori* SS1 or 26695, indicating the isolates tested possess the Lewis Y antigen.

## Reactivity of mAbs against *H. pylori* SS1 and 26695 in immunofluorescence

**Figure 4:**
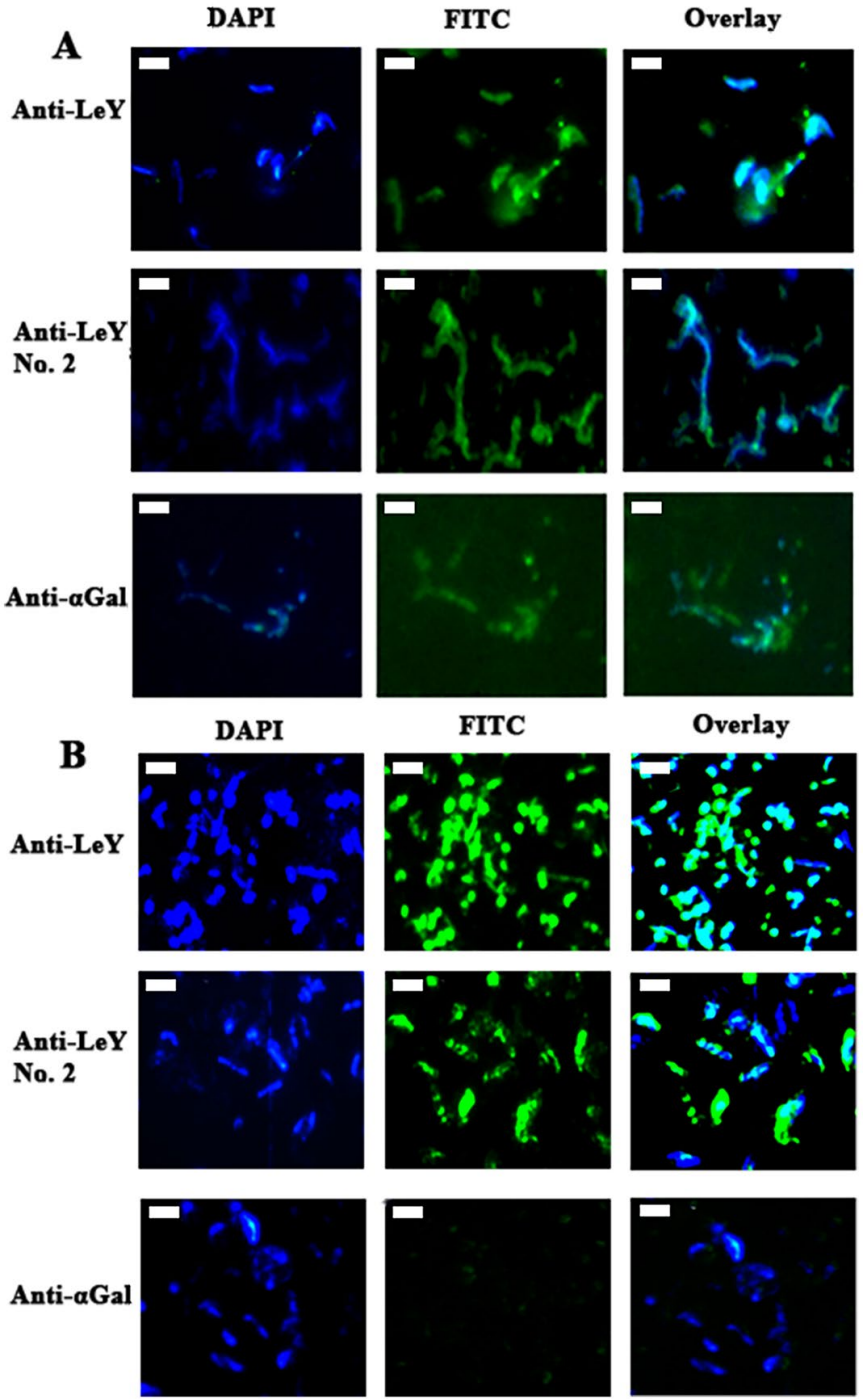
Immunofluorescence images of select Lewis antigen specific mAbs. (**A)** mAb binding to *H. pylori* SS1. (**B**) mAb binding to *H. pylori* 26695. The Le^y^ specific mAbs FG27 and LWY/1463 bound to intact SS1 and 26695 cells, confirming the presence of the Le^y^ antigen on *H. pylori* SS1 and 26695. The αGal specific mAb 15.101 binds the bacterial surface and also appears to stain the background, which may be due to bacterial protein from lysed bacterial cells. The white scale bar represents 2 µm. mAbs that recognise Le^a^/Le^x^ and Le^b^ were also tested and found not to bind to either *H. pylori* strain (Fig. S2).

The pattern of binding of the mAbs in IF was very similar to the results from the ELISA screen. For SS1 (Fig. 4A), the anti-Le^y^ mAbs bound to the bacteria and the anti-αGal mAb 15.101 also bound, although 15.101 appeared to have low reactivity in IF. Le^a^/Le^x^ and Le^b^ binding mAbs were also tested and showed no reactivity (Fig. S2). The binding pattern of Lewis antigen specific mAb to the 26695 strain (Fig. 4B) was also mostly consistent with both the ELISA and with the SS1 results. The binding of anti-Le^y^ mAbs to 26695 also indicates the presence of the Lewis Y antigen on this strain of *H. pylori*. The anti-αGal 15.101 showed almost no reactivity to 26695 via IF, indicating that the αGal determinants are mostly on intracellular glycoconjugates for this laboratory strain.

### Adhesion of mAbs and lectins measured using force spectrometry

Based on the results of the lectin and antibody screening, WGA and anti-Le^y^ antibodies were selected for AFM studies to investigate binding interactions with glycans on intact and viable *H. pylori* cells. AFM tips were functionalised by adsorption of either antibody or lectin to the tip and used to image *H. pylori* 26695. Data collected with functionalised AFM probes were then compared to data obtained using an uncoated tip. Anti-Le^y^ (mu3S193) and WGA were selected to be tested with the AFM technique based on the previously observed consistent binding in the immunoassays. The AFM is able to simultaneously measure the height of the bacterium against a flat background, and the adhesive force between the molecules used to functionalise the tip and the bacterium.

**Figure 5:**
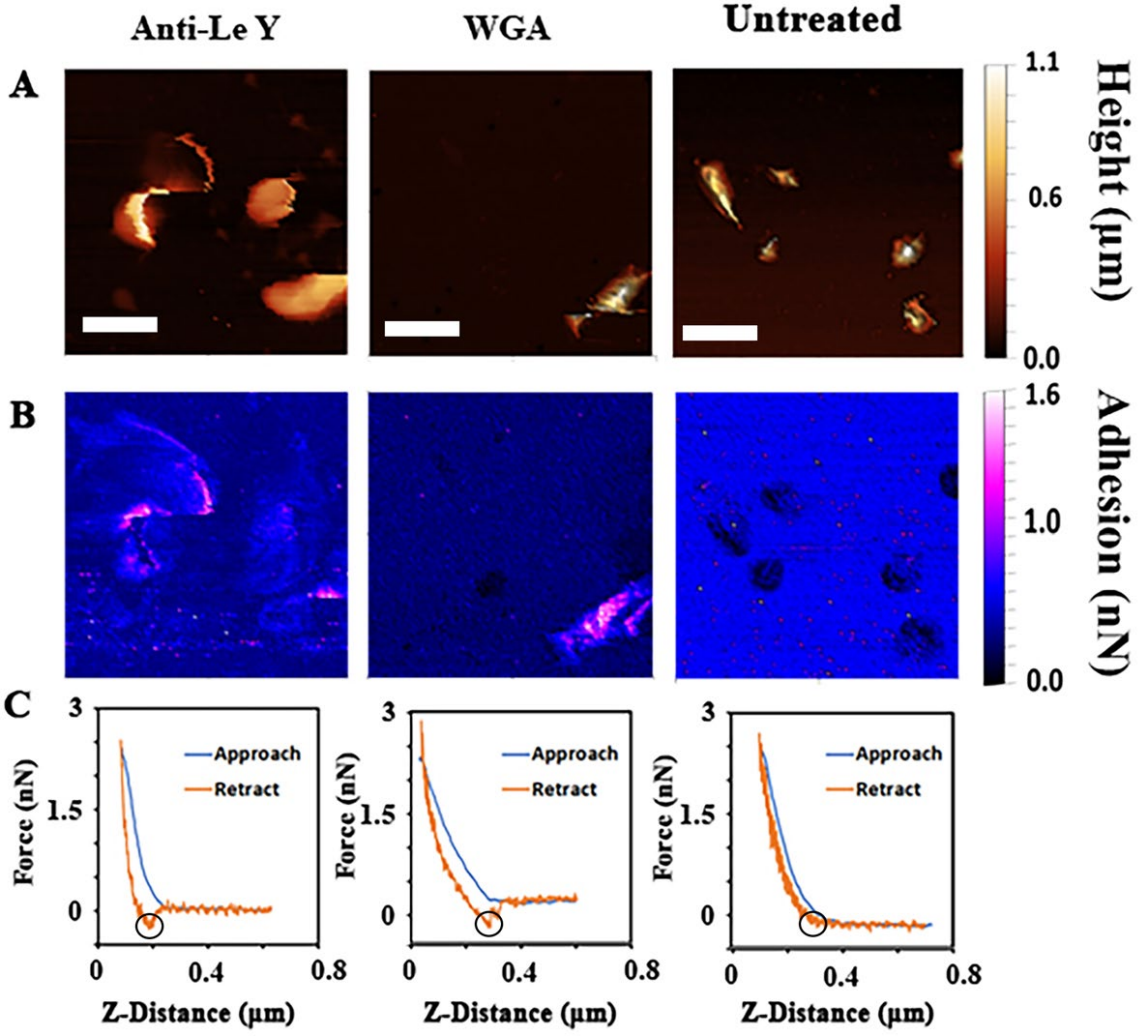
Comparison of adhesion between anti-Le^y^ and WGA treated tips and untreated tips. (**A**) The height of the bacterium above the surface scanned by the AFM. The bacteria were found to be 0.5–1.0 μm high, and to display a pleomorphic appearance. (**B**) The adhesive force spikes over the bacteria for both the anti-Lewis Y and the WGA treated tips while the untreated tip shows a lower adhesion than the background, illustrated by the false colour scale. (**C**) Representative adhesion force curves of a point of contact using the different tips. The retraction of the treated tip can be seen to spike in force for both anti-Le^y^ (left) and WGA (middle), which does not occur for the untreated tip (right). Maximum adhesion is indicated in each graph by the black circle. The white scale bars represent 1.5 μm.

Figure 5 shows AFM images and force profiles conducted with cantilevers functionalised with anti-Le^y^ and WGA, as well as untreated (as received) cantilevers. Here, images are produced in QI mode, which uses progressive force-mapping of the interface—The AFM tip is moved towards (approach) and away (retract) from the sample producing an image from the point of contact at each discreet X–Y surface location, while simultaneously collecting a force curve at each discreet location on the sample. This supplies topological (height) profiles of the interface (see Fig. 5A), as well as adhesion maps (see Fig. 5B). Tip-sample adhesion is measured in an AFM force profile as the minima of the force upon retraction from the sample, which represents the maximum force needed to remove the tip from the sample. Adhesion is observed in the retraction curve of an AFM force profile, as attractive forces between the AFM tip and the sample surface result in a negative force, reflecting the energy required to overcome molecular interactions during the separation of the tip and the sample. This force is then converted to a positive force of adhesion. This allows the measurement of the force (nN) of the binding events (Fig. 5C, minima highlighted by a circle). Together these measurements provide nanoscale detail of the adhesive interactions between antibodies or lectins and glycans present on *H. pylori* 26695. Adhesion events could be measured with functionalised tips but not with untreated tips. The anti-Le^y^ coated tip showed increased adhesion predominantly on and around the sides of the cells while WGA typically showed binding over the entire surface of the bacteria (Fig. 5B). Again, only the carbohydrate-binding protein coated cantilevers showed adhesion to the bacterial surfaces.

**Figure 6:**
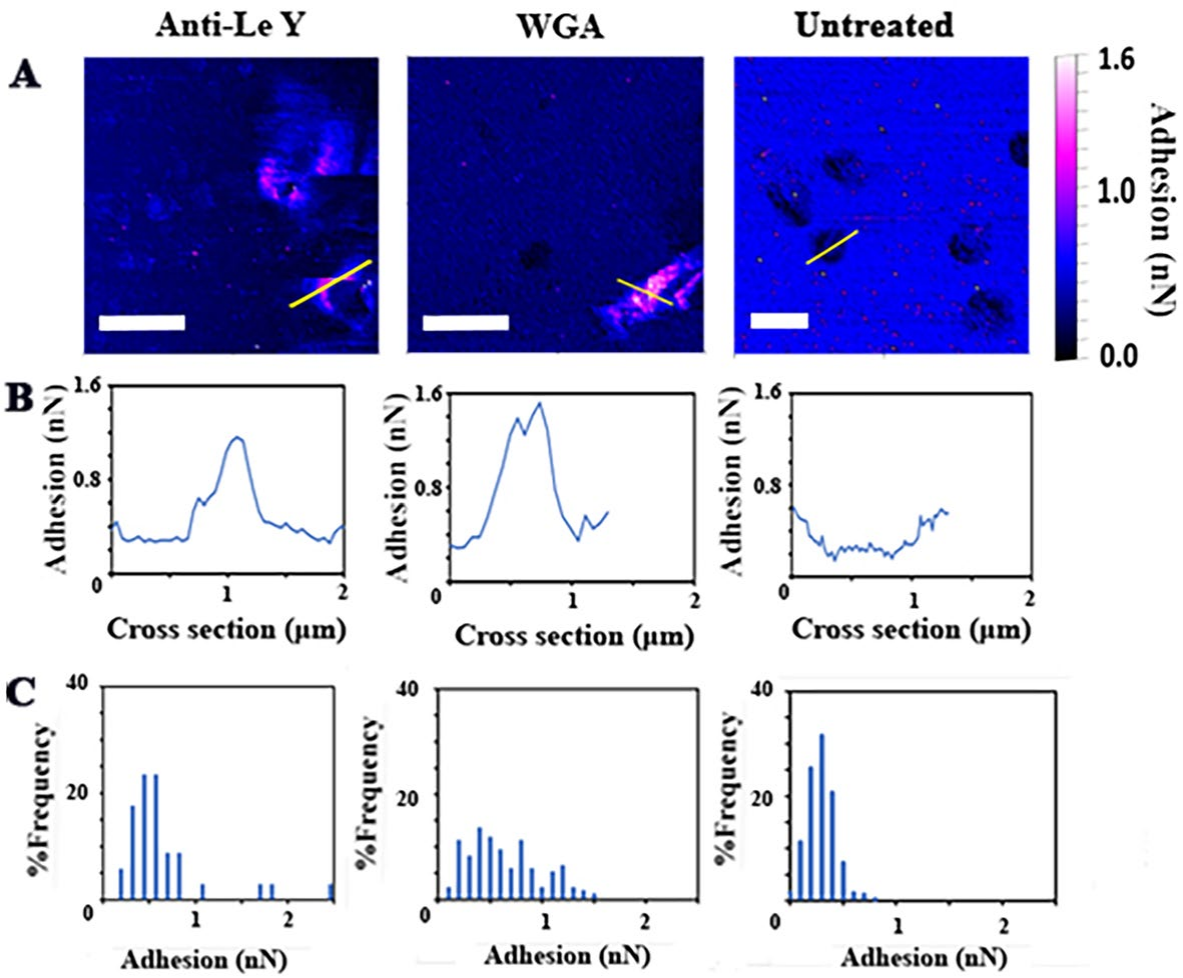
Cross section and adhesion events using treated and control tips on *H. pylori* 26695. Comparison of the adhesion profiles between an anti-Le^y^ or WGA treated tip, and an untreated tip. (**A**) Adhesion scans for treated and untreated tips. The yellow line shows where the cross-sectional data over individual bacteria was obtained. Scale bars represent 1.5 μm. (**B**) Cross-sectional adhesion data, demonstrating how the adhesion fluctuates as the AFM passes over the bacteria. (**C**) Frequency of adhesion events of the treated tips in comparison to the untreated tip obtained only from bacterial surfaces. The range of adhesion values can be seen to increase for treated tips, additionally a higher percentage of events over 0.5 nN can be seen for both treated tips with events over 1 nN occurring. This is in comparison to the untreated tip where over 90% of events occurred below 0.5 nN and all fell under 1.0 nN.

A cross section of adhesion data for a single bacterial cell was made for the anti-Le^y^ and WGA treated tips in comparison to an untreated tip (Fig. 6). These were able to demonstrate the increase in adhesion of both the lectin and mAb over the surface of the bacterial cell, where in comparison a plain AFM tip shows a reduction in adhesion over the softer surface of the bacterial cell. Furthermore, the height measurement was used to extract the adhesion event percentage frequencies across only bacterial cells. This allowed for a direct comparison of the force data between the treated tips and the untreated tip. Both treated tips displayed multiple adhesion events greater than 1.5 nN, in comparison to the untreated tip which had over 90% of readings below 0.5 nN and all readings were 1.0 nN and below. This result was reproducible with both anti-Le^y^ and WGA treated tips used on 3 separate occasions. The increase in the overall adhesion and the spike in adhesion events allow us to map the expression of the carbohydrates on the surface of the bacterial cell. Particularly the wider distribution of *N-*acetylglucosamine across the surface of the cell can be seen via the WGA adhesion while Le^y^ can be seen around the edges of the cell in localised areas. This finding likely reflects a different surface distribution of Le^y^ determinants when compared to glycans containing *N-*acetylglucosamine residues.

Following the adhesion experiments with WGA and anti-Le^y^, we performed blocking studies to confirm the specificity of binding. For WGA, a 100 mM solution of *N*-acetylglucosamine was added to the liquid bacterial suspension at a 1:1 dilution (for a final *N*-acetylglucosamine concentration of 50 mM) and incubated for 30 min before screening. This process was repeated for anti-Le^y^ using a 100 mM solution of the Lewis Y tetrasaccharide (final concentration 50 mM). In both cases, the glycan solutions blocked the adhesion from the treated tip, greatly reducing the adhesion of the tip across the surface of the bacteria (Fig. S3). WGA exhibited higher background binding than the anti-Le^y^ mAb, however it was still overall lower than the unblocked lectin over the surface of the bacteria (Fig. S4).

### Blocking Le^y^ with mAb reduces ***H. pylori*** adhesion to AGS cells

**Figure 7:**
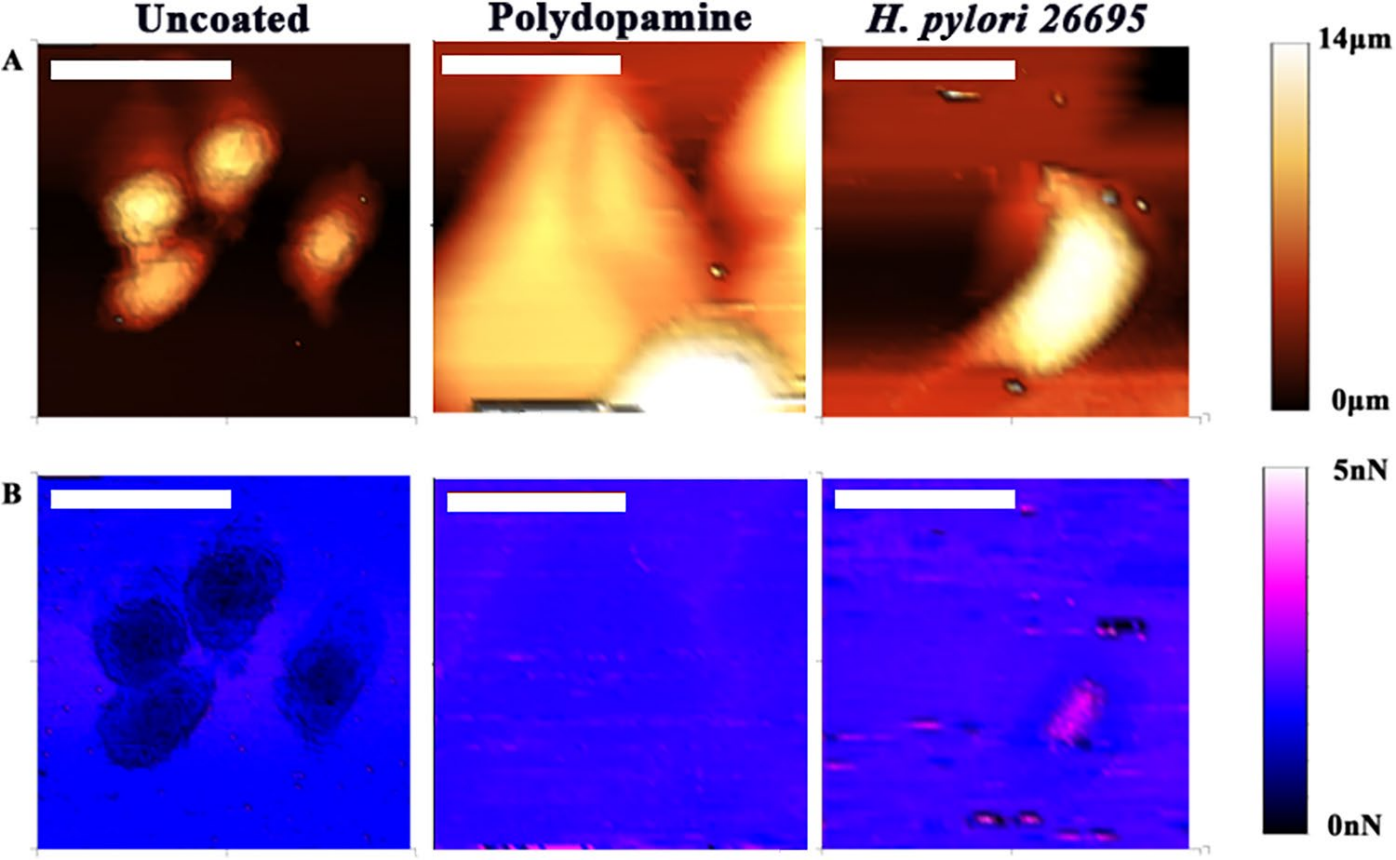
AFM imaging of AGS cells using a standard cantilever comparing an untreated tip to a tip coated with *H. pylori*. (**A**) shows the height measurement of AGS cells imaged by the AFM (left) a polydopamine coated tipless cantilever (centre) and the height measurement of AGS cells using a sharp tip coated with *H. pylori* (right) (**B**) shows the adhesion measurement of the standard untreated tip when probing an AGS cell (left), a polydopamine coated tipless cantilever (centre) and the adhesion of a *H. pylori* 26695 coated tip when probing an AGS cell (right). Increases in adhesion between *H. pylori* and the AGS cell can be seen towards the edges and in certain locations over the cell. The white scale bar represents 30 µm.

Once we had established the adhesion of anti-Le^y^ to *H. pylori* 26695 could be measured with AFM, we sought to demonstrate the adhesion of *H. pylori* 26695 to human gastric cells (AGS cells) and examine if the adhesion could be reduced by binding an anti-Le^y^ mAb to *H. pylori.* Figure 7 shows the AFM scan of AGS cells using a cantilever coated with *H. pylori* 26695 using polydopamine, in comparison to a plain AFM cantilever. The adhesion of the bacteria around the edges and towards the centre of the AGS cell can be clearly seen in comparison to the plain tip, which shows a reduction in adhesion over the softer cell surface.

**Figure 8:**
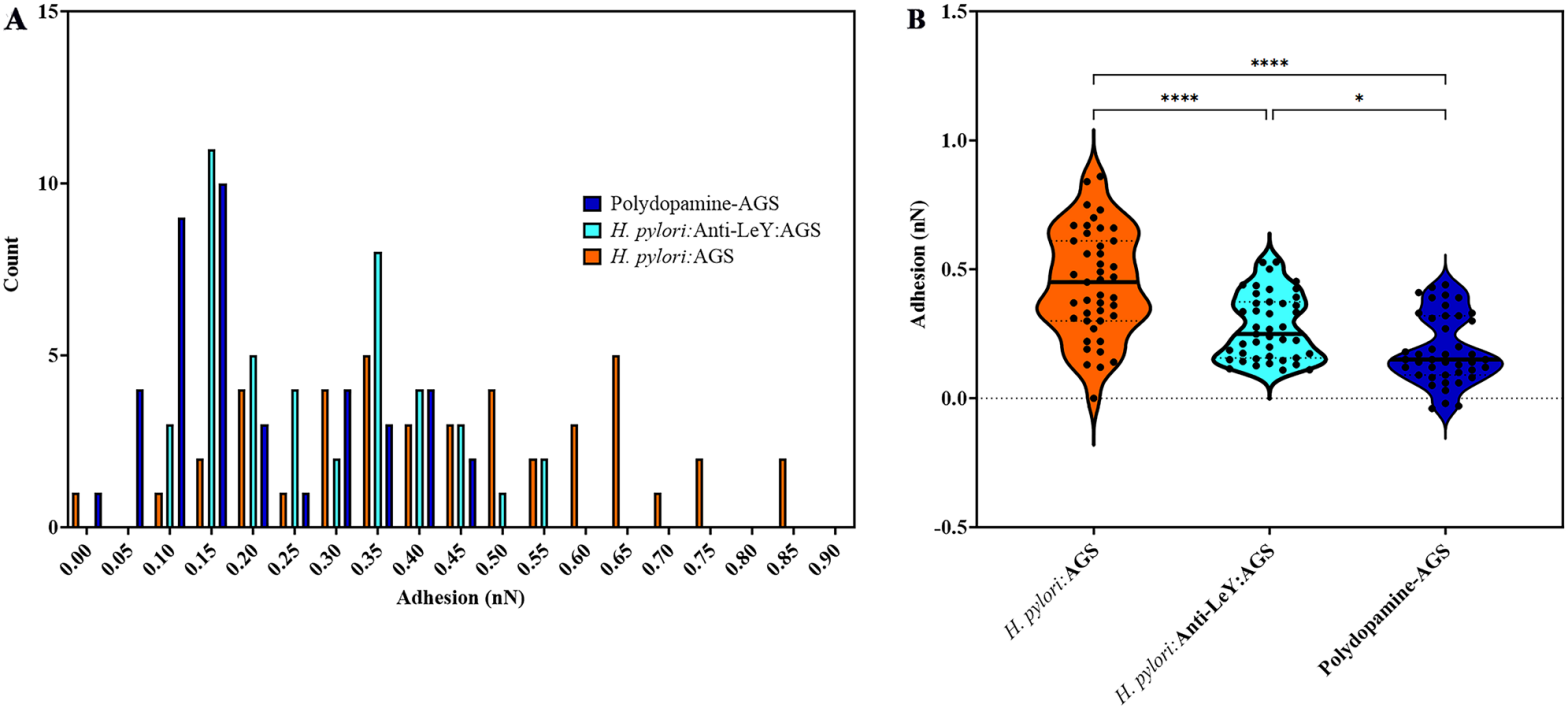
*Helicobacter pylori* binding to AGS cells and the effect of Le^y^ mAbs. (**A**) a histogram of the single point measurements of a *H. pylori* 26695 coated cantilever (orange) pressed against AGS cells compared to a *H. pylori* coated cantilever that has been incubated with anti-Le^y^ (cyan) and a polydopamine only cantilever (dark blue) (**B**) A violin plot of the same showing the distribution of the adhesion points and demonstrating the difference in the mean between the data sets. ****p value < 0.0001 and *p value < 0.05 by one-way ANOVA. The ANOVA summary can be found in Table S1, and the ANOVA multiple comparisons analysis can be found in Table S2.

A tipless AFM cantilever was then used to take adhesion measurements of *H. pylori* 26695 under three conditions. The first of which was the AFM cantilever coated with *H. pylori* 26695. Second, was using a cantilever coated only with polydopamine as a control. And finally, a cantilever coated with *H. pylori* 26695, that had been incubated with anti-Le^y^ for approximately 30 min. This was done to allow us to explore the potential of carbohydrate binding proteins to block the adhesion of *H. pylori* 26695 to AGS cells. In Fig. 8A a histogram of the adhesion under the three conditions can be seen, with the *H. pylori* coated cantilever displaying the highest adhesion of the three conditions (maximum ~ 0.85 nN), and with that the addition of anti-Le^y^ significantly reducing bacterial adhesion to the AGS cells (maximum 0.55 nN). Figure 8B shows a statistical comparison between the three conditions via one-way ANOVA, with a significant reduction in the mean adhesion of *H. pylori* 26695 to AGS cells on the addition of anti-Le^y^, indicating the ability of the carbohydrate binding mAb to interfere with bacterial adhesion.

## Discussion

Glycan expression by *H. pylori* is thought to play a role in both infection and evasion of the host immune system^35,36^. *Helicobacter pylori* has a known association to the Lewis tissue and histo-blood group antigens. Firstly, through adhesion proteins BabA and SabA which bind to Lewis antigens^37^, and secondly, through expression of Lewis determinants in *H. pylori* LPS that mimic Lewis antigens of the host, suggesting a role of these antigens in bacterial immune evasion^16,38^. Previous studies have characterised the glycan composition of the *H. pylori* LPS by molecular methods, structural methods, and immunological assays such as glycan microarrays and agglutination assays^14,19^. In a previous lectin microarray investigation, WGA, Con A, and RCA were shown to bind to the *H. pylori* 26695 strain^39^. Due to the complex role of glycans in *H. pylori* infection further studies are needed to better understand their role in the host–pathogen interaction. Some previous studies have looked at adhesion of bacterial cells using AFM, with El-Kirat-Chatel et al. using antibody treated tips to study *Pseudomonas fluorescens* large adhesion protein (LapA) and the relationship of said protein to the formation of biofilms, with LapA footprints recorded to be 50–600 pN^40^. Another study by Herman-Bausier et al. examined the adhesion of *Staphylococcus aureus* collagen binding protein Cna which demonstrated binding forces of ~ 1.2 nN to collagen, which was disrupted by multiple mAbs, including 9G7, C2 and 11H11^41^. Some studies have explored using AFM to measure bacterial glycan interactions with mAbs, lectins^42–44^, and, with regard to *H. pylori*, Parreira et al. demonstrated the mechanistic binding method between *H. pylori* blood-group binding adhesin (BabA) and Lewis B^29^. Parreira et al. also highlighted the potential for AFM in the development of new targeted therapies against bacteria^28^. As *H. pylori* glycans are known to play a role in bacterial adhesion to human cells, further exploration of how to interrupt glycan expression and binding by *H. pylori* may provide new targets for therapeutic development.

In this study, lectins, and glycan/Lewis targeting antibodies were used to profile the glycans of *H. pylori* strains SS1 and 26695 using ELISA with bacterial lysates, and IF on intact bacteria. Additionally, the interactions of anti-Le^y^ mAb and WGA with *H. pylori* were examined using AFM and followed up by the examination of the direct interaction between *H. pylori* 26695 and AGS cells. Furthermore, adhesion was shown to be disrupted by antibody-targeting of bacterial surface carbohydrates. The *H. pylori* LPS has been previously found to express a number of different structures, including an array of Lewis determinates at its nonreducing end^38^. In our study, WGA, Con A and anti-Le^y^ mAbs showed reactivity in both ELISA and IF, and sWGA showed reactivity in IF. The sialic acid recognising EBL was found to bind 26695 in ELISA. *Helicobacter pylori* has been previously found to interact with sialyl-Le^x^ antigens via the sialic acid binding adhesin (SabA), and glycosylation of *N*-acetylglucosamine (GlcNAc) structures with sialic acid or fucose residues^10,45,46^. Here, the 15.101 mAb, which binds to galactose-α1,3-galactose containing glycans was found to interact with 26695 in ELISA^47^. Notably, neither EBL nor 15.101 mAb showed reactivity in IF assays, which could possibly be explained by a difference in sensitivity between the two assays^48^. Alternatively, lack of binding in IF may suggest that the target glycans are internal and not displayed on the bacterial surface.

The lectin sWGA showed reactivity by IF but not by ELISA, and it is possible that the GlcNAc structure recognised by sWGA is disturbed during the lysis process or that it is present in low abundance. In the case of *H. pylori* SS1, WGA, ECA and UEA all bound in the ELISA and had been previously identified^49–51^. Con A also bound to SS1 glycans in both assays which is consistent with results for 26695. This differs from a previous study, where agglutination assays could not demonstrate a Con A interaction with SS1^14^. However, Con A interaction has been reported for clinical isolates of *H. pylori*, and it is possible that differences in assay sensitivities explain the observed differences^39,52,53^. Notably, several additional lectins were found to interact with SS1 compared to 26695, including PSA, which binds mannose, PHA-E, which binds biantennary glycosylated *N*-glycan bisecting GlcNAc, and EBL which binds sialic acid^54–56^. Most of these additional lectins that bound in ELISA did not bind via IF suggesting that these glycan targets are internal rather than displayed on the bacterial surface^57^. This may be the case for stored sugars, those currently undergoing biosynthesis, or may involve glycosylated proteins and enzymes^58^. Interestingly, mass spectrometry has revealed that *H. pylori* possess a vast array of glycoproteins expressed in different locations, including the cytoplasm, periplasm, and inner cell membrane, with as many as 52% of these glycoproteins expressed in the cytoplasm^59^*.*

The 15.101 mAb, an anti-αGal^30^, bound both in ELISA and IF for SS1 but appeared to have some background staining in the IF assay. In the case of SS1, while an αGal antibody has not been tested with SS1 before, lectin BS-I, which binds to αGal, was found to interact with SS1 in agglutination assays^14^. A previous study has also found that 26695 has a reduced variety of carbohydrate expression in comparison to clinical isolates^50^. Additionally, the difference in ELISA and immunofluorescence results, even within the same strain, highlights the likely differences that can be seen between internally and externally expressed glycan epitopes. While these observations have been raised previously^57^, they are important to note when profiling glycan expression.

AFM was used to image *H. pylori* and the nanoscale scans revealed the spiral bacillus morphology of the bacterium. A degree of pleomorphy was observed, as has previously been seen in *H. pylori* in light microscopy^60^. Bacterial flagella could only be sporadically seen on the scans, likely due to the regulation of flagella expression by *H. pylori* on solid media^61^*.* The AFM analysis also provided a large quantity of force data, allowing determination of the interaction between *H. pylori*, and anti-Le^y^ and WGA*.* Anti-Le^y^ could be seen to interact with *H. pylori* along the sides of the bacteria and on flagella-like objects where those could be seen, with the force of adhesion ranging between 1 and 2.5 nN (Fig. 5A). The adhesion pattern observed for WGA indicates more surface area of the bacteria was coated with *N*-acetylglucosamine glycans, with WGA:glycan adhesion forces ranging between 1 and 2 nN. This contrasts with measurements taken with the nontreated AFM tip, which frequently showed lower adhesion than the background and had no data points over 1 nN (Fig. 6).

In this study, we also explored the ability of mAbs against carbohydrates to block adhesion of bacteria to stomach cells. We observed a significant reduction in adhesion of *H. pylori* 26695 to AGS cells after binding of an anti-Le^y^ mAb to a bacteria-coated tip, which reduced the mean adhesion by 0.16 nN and the maximum adhesion by 0.29 nN. This finding supports the potential of carbohydrate targets for future development of therapeutics for *H. pylori* infection. The work presented here differs from previous AFM studies on *H. pylori* and is the first study focusing on the characterisation and quantification of antibody-glycan interactions and blocking with an anti-Le^y^ mAb of *H. pylori* 26695 adhesion to AGS cells in vitro.

While the current investigation focuses on the surface glycans and targeting of *H. pylori* with Le^y^ binding antibodies, the glycan-mediated host interactions are complex. Effective in vivo blocking of *H. pylori* interactions with gastric mucosa and gastric epithelial cells will need to consider several factors. As previously mentioned, *H. pylori* has several carbohydrate binding OMPs that are involved in gastric adhesion. The most predominant of these, BabA, binds to the Le^b^ and H1 blood group antigens. Glycan recognition by BabA is mediated by a terminal α1,2 linked fucose (Fuc) on a Galβ1-3GlcNAc core, allowing it to commonly adhere to Le^b^ positive epithelial cells and MUC-5AC in the mucosa^62,63^. Notably, BabA does not bind to Le^a^ that lacks an α1,2-linked Fuc residue^5^. Furthermore, loss of fucosyltransferase 2 (FUT2) or non-secretion of Le^b^ impairs the adherence of the bacteria to the gastric mucosa^64^. However, *H. pylori* possess other non-BabA mediated methods of adhesion, and expression of BabA may be modified by changing gastric conditions, and is lost entirely in some animal models^37^. The SabA protein binds preferentially to SLe^x^, but also binds to SLe^a^^37,65^. These glycans are minimally expressed in the gastric epithelium and mucosa in healthy individuals, but are upregulated in inflammatory and cancerous states, leading to the suggestion that SabA is important in chronic infection^7^. *Helicobacter pylori* may also play a role in the regulation of expression of host glycans in chronic infection ^66^. Another important OMP is HopQ, which binds carcinoembryonic antigen-related cell adhesion molecules (CEACAMs)^67^. In relation to HopQ specifically, CEACAM1, CEACAM5 and CEACAM6 are all upregulated in gastritis caused by chronic *H. pylori* infection, again suggesting a mechanism for *H. pylori* promoting its own persistence in an inflammatory environment^67,68^. Additionally, the LacdiNAc binding adhesin (LabA) or HopZ plays a potential supporting role in binding to MUC-5AC^69^ and the proteins AlpA and AlpB display a role in binding to host laminin^70^.

A motivation for this study, is *H. pylori* LPS has been shown to play a significant role in adhesion, and *H. pylori* mutants with truncated LPS exhibited reduced adhesion to AGS cells^21^. *Helicobacter pylori* employs a generalized two-step system for the glycosylation of adhesion proteins such as BabA, utilizing similar enzymes to what are responsible to LPS biosynthesis, with disruption of this process resulting in a loss of bacterial adhesion in vitro^71^. The complexity of the mechanisms of adhesion of *H. pylori* underlines the significance for the survival and persistence of the bacterium. Interference of *H. pylori* interactions with gastric carcinoma cells by monoclonal antibodies specific for Le^y^ glycans as demonstrated here is one further step in understanding the complex roles of carbohydrates in bacteria–host interactions.

## Conclusion

This study explored glycan-protein interactions of two model *H. pylori* laboratory strains and examined the molecular adhesion of lectin WGA and anti-Le^y^ to *H. pylori* 26695 using AFM. We have identified several glycan-binding proteins that interact with two reference strains of *H. pylori,* highlighting the differences in glycan expression between these strains and emphasising the usefulness of AFM force spectroscopy in measuring the interactions between anti-glycan proteins and target ligands on live bacterial cells. Furthermore, this study demonstrated a reduction in *H. pylori* adhesion to AGS cells via carbohydrate-targeting antibodies, successfully blocking adhesion of *H. pylori* 26695 to AGS cells after incubation with anti-Le^y^ mAb via AFM measurements.

### Supplementary Information

Supplementary Information 1.

## Data Availability

All data are included in the Supplemental Information or available from the corresponding author upon reasonable request. The raw numbers for charts and graphs are available in the Source Data file provided with this paper.
