# Supplementary material for: Probing the expression and adhesion of glycans involved in Helicobacter pylori infection
Source: Sci Rep. 2024 Apr 13;14:8587. doi: 10.1038/s41598-024-59234-w (PMC11016089; doi:10.1038/s41598-024-59234-w)
Supplement: Supplementary file 1 — Supplementary Information 1. [file 41598_2024_59234_MOESM1_ESM.pdf]

Supporting Information

Probing the expression and adhesion of glycans involved in *Helicobacter pylori* infection

Daniel Sijmons[1], Simon Collett[1,2], Caroline Soliman[1,3], Andrew J Guy[1,4], Andrew M Scott[5,6], Lindy G Durrant[7,8], Aaron Elbourne[1], Anna K Walduck[1,9,*] & Paul A Ramsland[1,10,11,*]

[1]School of Science, RMIT University, Melbourne, Victoria 3000, Australia
[2]Department of Paediatrics, The University of Melbourne, Parkville, Victoria 3010, Australia
[3]Department of Microbiology and Immunology, Peter Doherty Institute for Infection and Immunity, The University of Melbourne, Melbourne, Victoria 3000, Australia
[4]ZiP Diagnostics, Collingwood, Victoria 3066, Australia
[5]Olivia Newton-John Cancer Research Institute and School of Cancer Medicine, La Trobe University, Melbourne, Victoria, Australia
[6]Department of Molecular Imaging and Therapy, Austin Health and Faculty of Medicine, The University of Melbourne, Melbourne, VIC, Australia
[7]Scancell Limited, University of Nottingham Biodiscovery Institute, Nottingham, UK
[8]Division of Cancer and Stem Cells, School of Medicine, University of Nottingham Biodiscovery Institute, Nottingham, UK
[9]Rural Health Research Institute, Charles Sturt University, Orange, New South Wales 2800, Australia
[10]Department of Immunology, Monash University, Melbourne, Victoria 3004, Australia
[11]Department of Surgery, Austin Health, The University of Melbourne, Heidelberg, Victoria 3084, Australia

*Co-corresponding authors: anwalduck@csu.edu.au and paul.ramsland@rmit.edu.au

Materials Included

Figure S1 Lectins that did not demonstrate binding to *H. pylori*

Figure S2 mAbs that did not demonstrate binding to *H. pylori*

Figure S3 Blocking of anti-Le^y and WGA treated tips

Figure S4 Comparison of treated tips with and without glycan blocking solutions

Table S1 ANOVA summary of *H. pylori* 26695 interaction with AGS cells and blocking with anti-Le^y

Table S2 One-way ANOVA multiple comparisons analysis of *H. pylori* 26695 binding to AGS cells and blocking with anti-Le^y

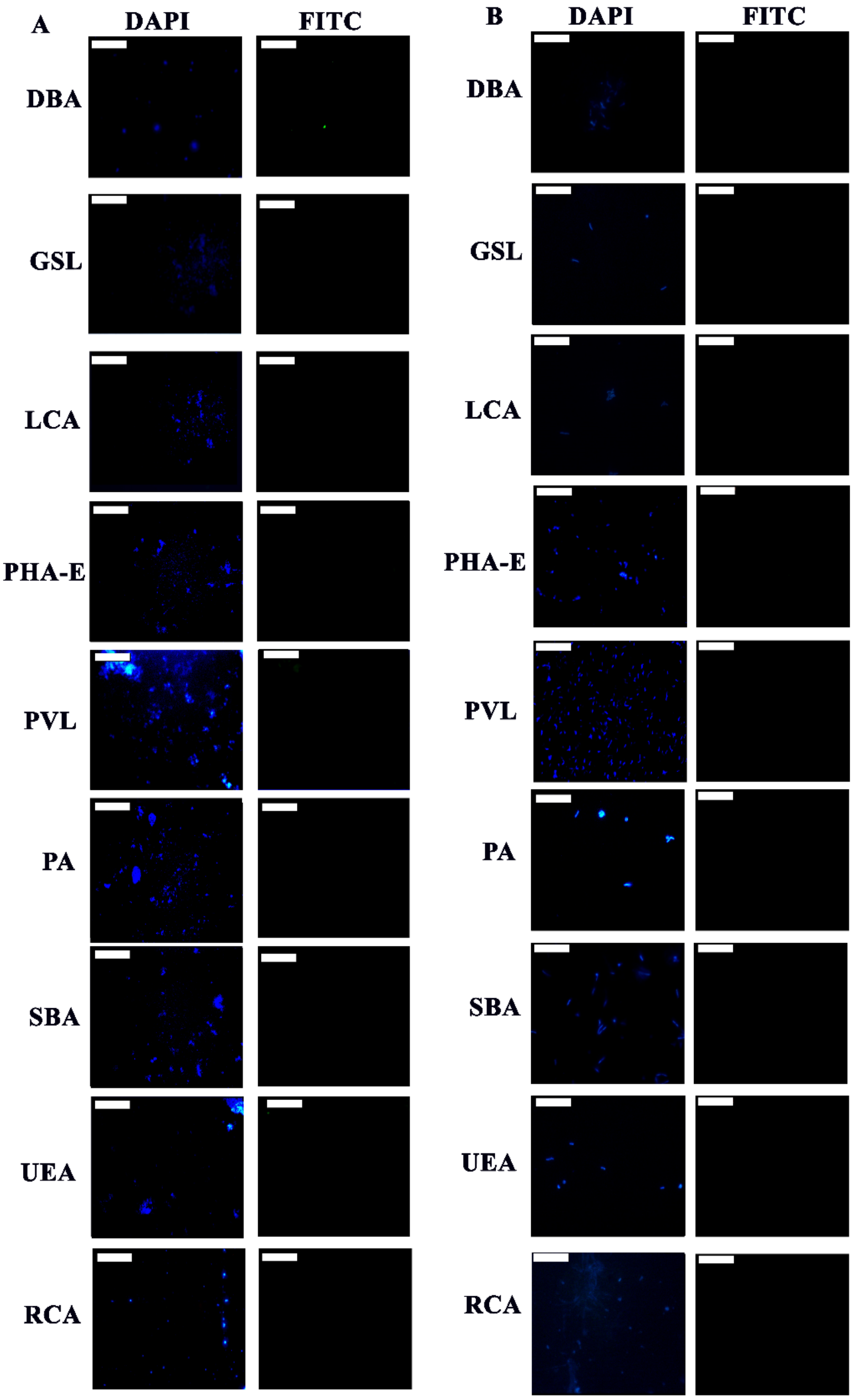

Figure S1 Lectins that did not demonstrate binding to *H. pylori* (**A**) Lectins that did not bind to SS1, the bacteria nucleic acid is labelled blue with DAPI (left) and the lectins green with FITC (right). (**B**) Lectins that did not bind to 26695, the bacteria nucleic acid is labelled blue with DAPI (left) and the lectins green with FITC (right). The white scale bar represents 10 µm.

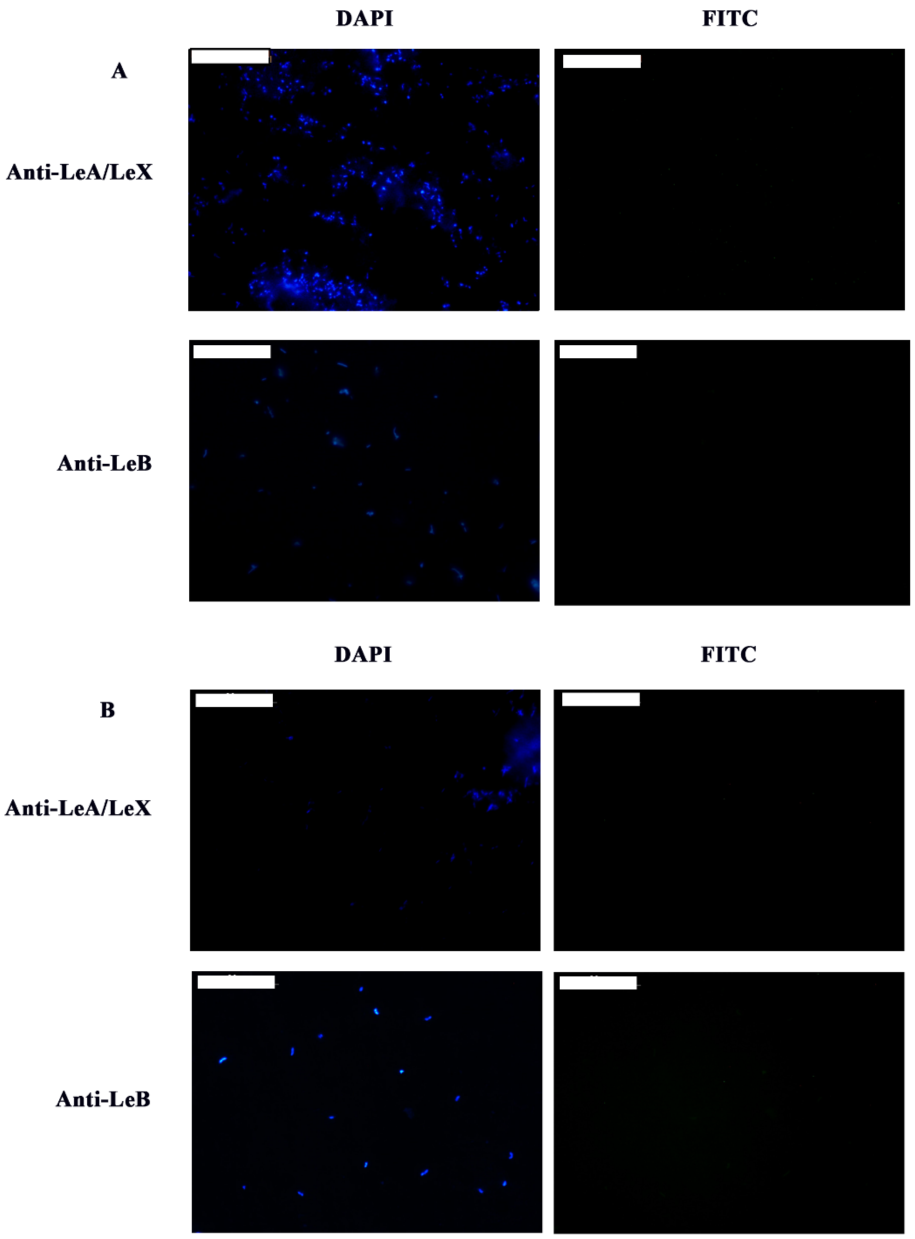

Figure S2 mAbs that did not demonstrate binding to *H. pylori* (A) mAbs that did not bind to SS1, the bacteria nucleic acid is labelled blue with DAPI (left) and the mAbs green with FITC (right). (**B**) mAbs that did not bind to 26695, the bacteria nucleic acid is labelled blue with DAPI (left) and the mAbs green with FITC (right). The white scale bar represents 10 µm.

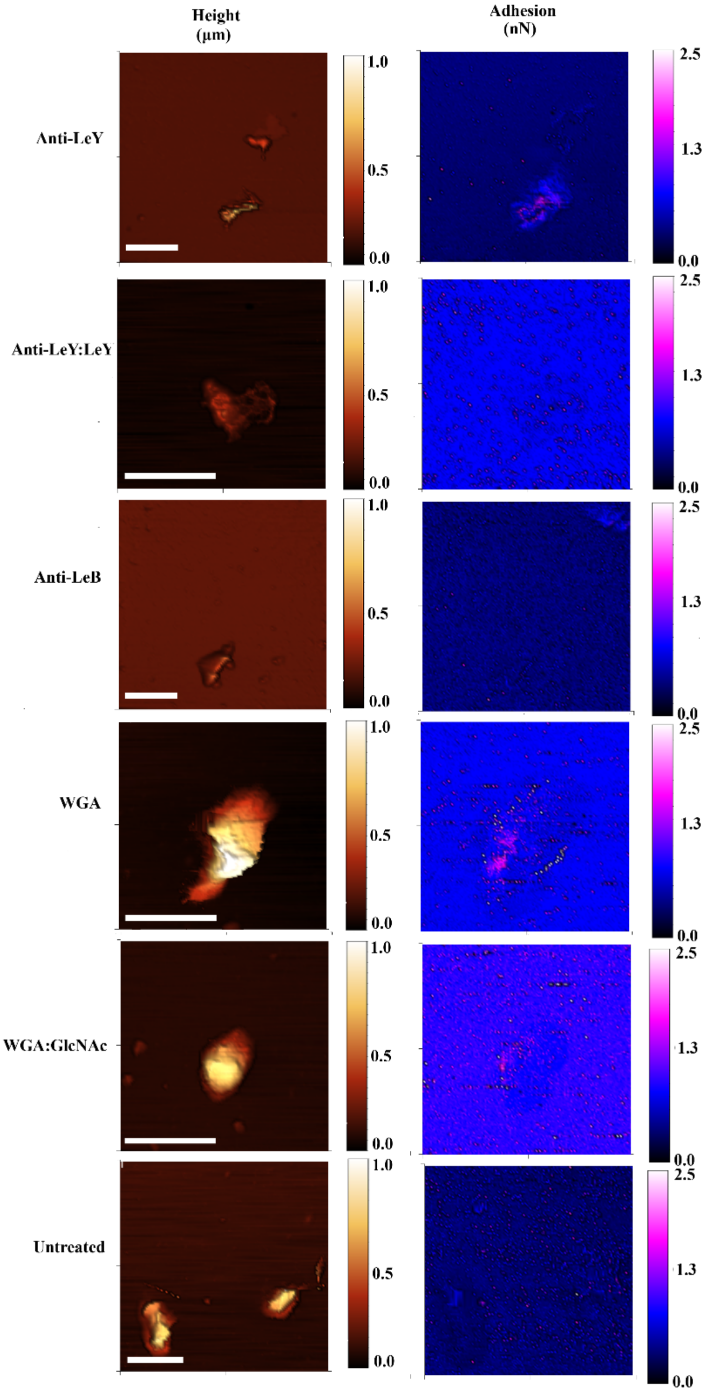

Figure S3 Blocking of anti-Le^y and WGA treated tips. A comparison of the adhesion of the treated AFM tips over the bacteria compared to when a 100 mM glycan solution is added. The white scale bar represents 1.5 µm. Increased adhesion can be seen where anti-Le^y and WGA treated tips are used respectively, when Le^y and GlcNAc are added respectively this specific adhesion over the bacteria no longer spikes, and the background adhesion is raised. Anti-Le^b treated and untreated tip show no adhesion.

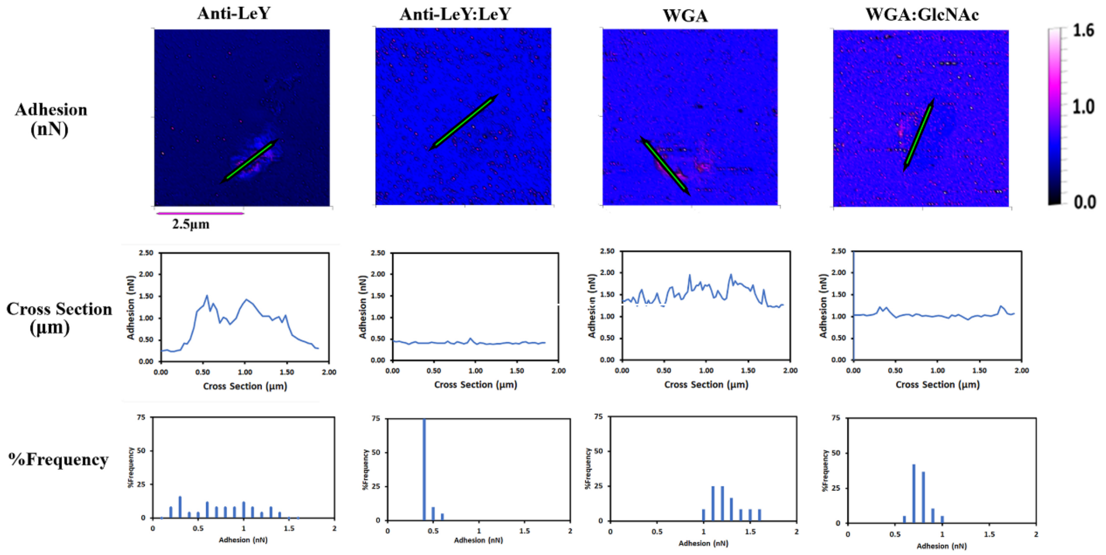

Figure S4 Comparison of treated tips with and without glycan blocking solutions. Anti-Le^{y} and WGA treated tips appear to have raised adhesion, supported by the cross section and frequency bar graph, these can then be seen to be reduced when the glycan blocking solutions are introduced, however the WGA adhesion is noticeably higher than the background overall, likely due to a more adhesive environment with the introduction of the GlcNAc solution.

Table S1 ANOVA summary of *H. pylori* 26695 interaction with AGS cells and blocking with anti-Le^y

**ANOVA Summary**	
F	28.60
P value	<0.0001
Significant diff. among means (P < 0.05)?	Yes
R squared	0.3122

Table S2 One-way ANOVA multiple comparisons analysis of *H. pylori* 26695 binding to AGS cells and blocking with anti-Le^{y}

Test details	Mean 1	Mean 2	Mean Diff.	SE of diff	Adjusted P Value
H. pylori:AGS vs. *H. pylori*:Anti-LeY:AGS"	0.4449	0.2765	0.1684	0.03447	<0.0001
H. pylori:AGS vs. Polydopamine-AGS"	0.4449	0.1884	0.2565	0.03447	<0.0001
H. pylori:Anti-LeY:AGS vs. Polydopamine-AGS"	0.2765	0.1884	0.08814	0.03447	0.0313
